# *Tropheryma whipplei*, the Agent of Whipple's Disease, Affects the Early to Late Phagosome Transition and Survives in a Rab5- and Rab7-Positive Compartment

**DOI:** 10.1371/journal.pone.0089367

**Published:** 2014-02-24

**Authors:** Giovanna Mottola, Nicolas Boucherit, Virginie Trouplin, Abdoulaye Oury Barry, Philippe Soubeyran, Jean-Louis Mege, Eric Ghigo

**Affiliations:** 1 UMR MD2, Aix-Marseille University and IRBA, Bd P Dramard, Marseille, France; 2 Department of Molecular Medicine and Medical Biotechnology, University of Naples “Federico II”, Naples, Italy; 3 CNRS UMR 7278, IRD198, INSERM U1095, UM63, Aix-Marseille University, Marseille, France; 4 INSERM U1068, CNRS UMR7258, UM105, CRCM-Institut Paoli-Calmettes, Aix-Marseille University, Marseille, France; UPR 3212 CNRS -Université de Strasbourg, France

## Abstract

*Tropheryma whipplei*, the agent of Whipple's disease, inhibits phago-lysosome biogenesis to create a suitable niche for its survival and replication in macrophages. To understand the mechanism by which it subverts phagosome maturation, we used biochemical and cell biological approaches to purify and characterise the intracellular compartment where *Tropheryma whipplei* resides using mouse bone-marrow-derived macrophages. We showed that in addition to Lamp-1, the *Tropheryma whipplei* phagosome is positive for Rab5 and Rab7, two GTPases required for the early to late phagosome transition. Unlike other pathogens, inhibition of PI(3)P production was not the mechanism for Rab5 stabilisation at the phagosome. Overexpression of the inactive, GDP-bound form of Rab5 bypassed the pathogen-induced blockade of phago-lysosome biogenesis. This suggests that *Tropheryma whipplei* blocks the switch from Rab5 to Rab7 by acting on the Rab5 GTPase cycle. A bio-informatic analysis of the *Tropheryma whipplei* genome revealed a glyceraldehyde-3-phosphate dehydrogenase (GAPDH) homologous with the GAPDH of *Listeria monocytogenes*, and this may be the bacterial protein responsible for blocking Rab5 activity. To our knowledge, *Tropheryma whipplei* is the first pathogen described to induce a “chimeric” phagosome stably expressing both Rab5 and Rab7, suggesting a novel and specific mechanism for subverting phagosome maturation.

## Introduction

Bacteria are internalised by macrophages in phagosomes and transported to phago-lysosomes, where they are destroyed. However, bacterial pathogens have evolved multiple strategies to interfere with phago-lysosome biogenesis, allowing them to survive and replicate within their host cells, leading to the failure of the immune response [1], [2].

The transformation of phagosomes into phago-lysosomes involves the gradual acquisition of markers from the endosomal compartment. Rab5 and Rab7 are small GTPases that continuously shift from an active GTP-bound form, which is necessary for the recruitment of effectors, to an inactive GDP-bound form. Rab5 regulates the fusion events allowing the conversion from early to late phagosomes. Rab7 is required for the fusion events allowing the conversion from late phagosomes to phago-lysosomes. The progression from early to late compartments requires a transient phase of Rab5 and Rab7 coexisting together on the membranes, followed by Rab5 inactivation and a switch to Rab7 activity [3], [4].

Bacterial pathogens target Rab functions to create a compartment suitable for their replication in host cells [5]. For example, *Mycobacterium tuberculosis* blocks the maturation of mycobacterium-containing phagosomes at the Rab5-positive stage by inhibiting production of phosphatidylinositol-3-phosphate (PI(3)P) [6], [7], which is required for the recruitment of Rab5 and its effectors [8]. *Helicobacter pylori* induces the formation of a Rab7-positive vacuole, blocking maturation into phago-lysosomes [9], [10], and *Listeria monocytogenes* interferes with Rab5 activity, residing in a Rab5-positive early phagosome, before escaping into the cytosol [11]. Unravelling the nature of the compartment where pathogens localise is helpful for understanding their takeover mechanisms and the establishment of the infectious disease.

*Tropheryma whipplei* is an actinomycete responsible for a multi-systemic infection called Whipple's disease [12]. Without antibiotics, the course of Whipple's disease is fatal [13]. It has been demonstrated that the host cell of *T. whipplei* is the macrophage, in which it induces an M2 non-microbicidal program [14]. *T. whipplei* replicates in both macrophages and non-microbicidal cells, reaching a maximum replication rate at 12 days after infection, and resides in a phagosome unable to fuse with lysosomes [15], [16]. The mechanism underlying the blockade by *T. whipplei* of phago-lysosome biogenesis remains unknown.

Here, we purified and characterised from macrophages the intracellular compartment where *T. whipplei* localises, and we investigated the mechanism used by the Whipple's agent to inhibit phago-lysosome biogenesis. Our data show that *T. whipplei* affects the transition from early to late phagosomes by blocking the Rab5-to-Rab7 switch.

## Results

### Characterisation of the intracellular compartment containing *T. whipplei*

**Figure 1:**
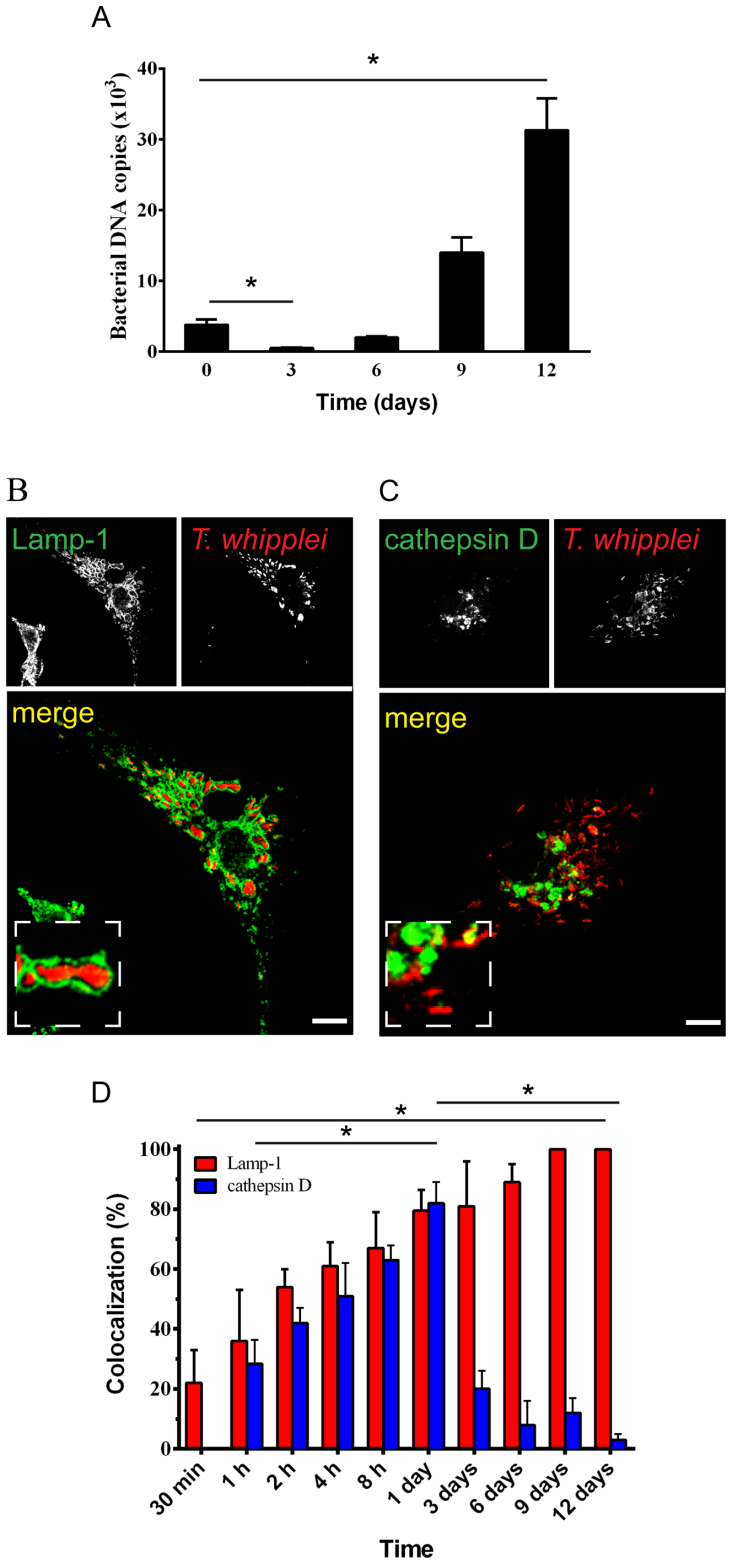
*T. whipplei* survives and replicates in a Lamp-1- but not cathepsin D-positive compartment within BMDMs. (**A**) BMDMs were incubated with *T. whipplei* (bacterium-to-cell ratio of 50∶1) for 4 hours (day 0). Cells were then washed to remove free bacteria and incubated for additional periods (days). The copy number of bacterial DNA was determined by qRT-PCR. The results are expressed as the mean ± SD from 3 experiments (*p<0.05). (**B–D**) The colocalisation of *T whipplei* with either (**B**) Lamp-1 or (**C**) cathepsin D was analysed in BMDMs by immunofluorescence and confocal microscopy. The percentage of *T whipplei* colocalising with either Lamp-1 or cathepsin D was quantified over the time (**D**). The results are expressed as the mean ± SD from 3 experiments (*p<0.05). The scale bars indicate 5 µm.

Bone marrow-derived macrophages (BMDMs) were infected with *T. whipplei*, and the intracellular fate of the bacteria was followed for 12 days. *T. whipplei*, after a transient phase of elimination at day 3, replicated within macrophages (**Figure 1A**) as previously described [15]. The intracellular localisation of *T. whipplei* was evaluated by immunofluorescence and confocal microscopy. Consistent with previous results [15]–[17], we observed that the *T. whipplei* compartment is surrounded by Lamp-1, a protein marker for late phagosomes and phago-lysosomes (**Figures 1B and 1D**). At 30 minutes post-infection, 22±11% of phagosomes containing *T. whipplei* colocalised with Lamp-1; this percentage increased progressively and reached 61±8% at 4 hours post-infection. By 9–12 days after infection, all detected bacteria were surrounded by Lamp-1 (**Figure 1D**). A time-course study of the colocalisation of *T. whipplei* with cathepsin D, a lysosomal enzyme, showed two successive phases (**Figures 1C and 1D**). At 1 hour after infection, 28.4±8% of phagosomes containing *T. whipplei* acquired cathepsin D. This percentage increased, reaching a maximum value after one day (82±7%). This phase of cathepsin D acquisition by *T. whipplei* phagosomes is correlated to the elimination of the majority of organisms by BMDMs, as previously shown (**Figure 1A** and [15]). A minor fraction of bacteria was able to replicate beginning on day 6 and did not colocalise with cathepsin D **(** **Figure 1D** and [15]). Indeed, the percentage of *T. whipplei* phagosomes containing cathepsin D sharply decreased on day 3 and had almost disappeared on day 12 (**Figures 1C and 1D**).

Consistently with previous results [15]–[17], these data show that the replicating fraction of the bacteria localises within a Lamp-1-positive late phagosome unable to fuse with lysosomes.

### Purification of the intracellular compartment containing *T. whipplei*

**Figure 2:**
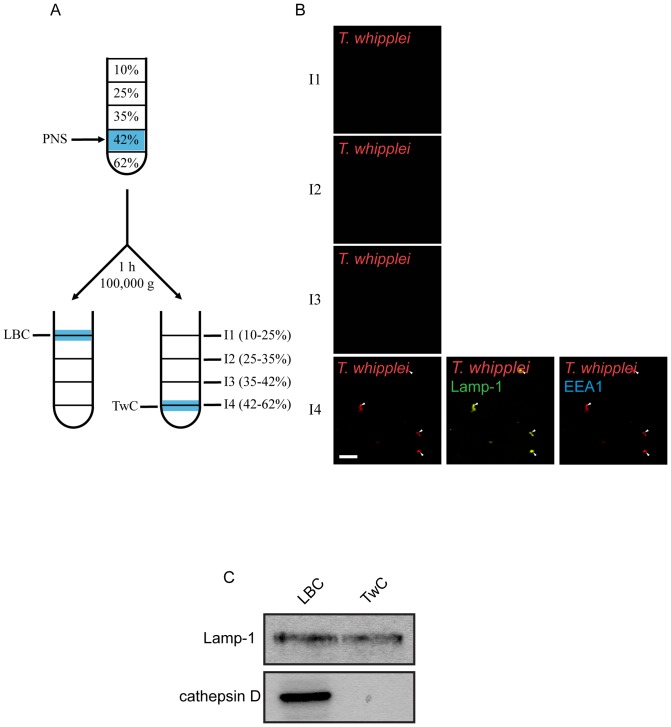
Purification of the *T. whipplei*-containing compartment from BMDMs. (**A**) Schematic view of the purification procedure. The compartments containing latex beads (LBC) or *T. whipplei* (TwC) were recovered in fractions I1 and I4 of the sucrose gradient, respectively. (**B**) All the sucrose gradient fractions (fractions I1 to I4) were analysed by immunofluorescence (n = 4) for the presence of *T. whipplei* (red), EEA1 (blue) or Lamp-1 (green). The scale bars indicate 5 µm. (**C**) Purified LBC and TwC fractions were analysed by western blot for the presence of Lamp-1 and cathepsin D. Immunoblot representative of 3 experiments.

To more fully characterise the compartment containing *T. whipplei* and understand the blockade of phago-lysosome biogenesis, we purified 12-day-old phago-lysosomes containing either latex beads (latex beads compartment, LBC) or *T. whipplei* (*T. whipplei* compartment, TwC) by ultracentrifugation on a sucrose gradient. LBC was recovered and collected at the 10 to 25% sucrose interface, as previously described (**Figure 2A**) [18], [19]. The sucrose fraction containing bacteria was identified by immunofluorescent detection of *T. whipplei*. *T. whipplei* organisms were recovered at the 42 to 62% sucrose interface (I4) (red, **Figure 2B**). The presence of contaminant endosomes or lysosomes in the 42 to 62% sucrose interface (I4) was investigated using antibodies against anti-EEA1 (Early Endosome Auto-antigen), which detects early endosomes and early phagosomes, and anti-Lamp-1, which detects late endosomes, late phagosomes and lysosomes. As shown above, TwC was positive for Lamp-1 but not for EEA1, confirming that it is a late phagosome. Moreover, the I4 fraction did not contain early endosomes or late endosomes because single-staining of either EEA1 or Lamp-1 was not detected (blue or green, **Figure 2B**). The quality of our phagosome preparation was also verified by western blotting for the detection of Lamp-1 and cathepsin D (**Figure 2C**). Both LBC and TwC were positive for Lamp-1. However, in contrast to LBC, TwC did not contain cathepsin D.

These data confirmed that *T. whipplei* does not reside in phago-lysosomes but in late phagosomes.

### *T. whipplei*-containing phagosomes express Rab5 and Rab7 GTPases

**Figure 3:**
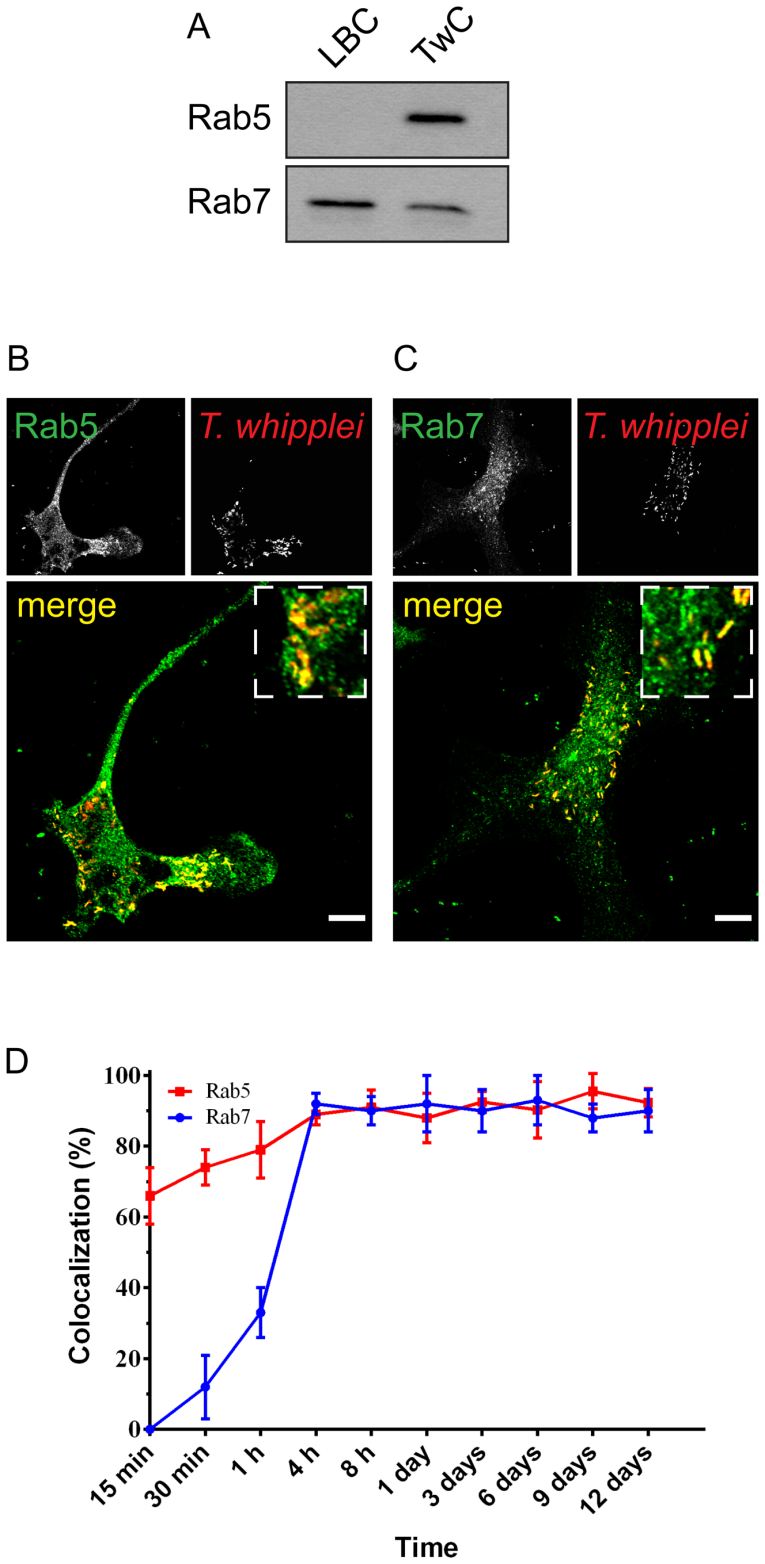
*T. whipplei* phagosomes continuously harboured both Rab5 and Rab7. (**A**) Purified LBC and TwC fractions were analysed by western blot for the presence of Rab7 and Rab5. Immunoblot representative of 3 experiments. (**B–C**) The colocalisation of *T whipplei* with Rab5 (**B**) or Rab7 (**C**) was analysed by immunofluorescence and confocal microscopy in BMDMs at day 12 after infection (n = 3). The scale bars indicate 5 µm. (**D**) The percentage of *T whipplei* colocalising with either Rab5 or Rab7 was quantified over time. The results are expressed as the mean ± SD from 3 experiments (*p<0.05).

The small GTPases Rab5 and Rab7 are key regulators of endosome and phagosome maturation and are necessary for phago-lysosome biogenesis [20]. Phago-lysosome biogenesis requires a transition from a Rab5-positive phagosome (early phagosome) to a Rab7-positive phagosome (late phagosome), which then matures into a phago-lysosome. Rab5 and Rab7 transiently coexist on endosomes to allow progression from early to late endosomes [21]. Because the phagosome containing *T. whipplei* is unable to fuse with lysosomes, we decided to verify whether there was any defect in the expression level of Rab5 and/or Rab7 or in their recruitment to the phagosome membrane. No variation in the amount of Rab5 and Rab7 mRNA was detected in infected cells (**Figure S1**). Next, Western blot analysis was performed on purified TwC to evaluate the presence of Rab5 and Rab7 (**Figure 3A**). As expected, when phagosome maturation occurs, Rab7, but not Rab5, was detected in the LBC fraction [22]. Surprisingly, in the TwC fraction, both Rab7 and Rab5 were present (**Figure 3A**). Consistently, immunofluorescence and confocal microscopy analysis on BMDMs at day 12 post-infection showed that the phagosome containing *T. whipplei* is positive for both Rab5 (**Figure 3B**) and Rab7 (**Figure 3C**). The time-course of the presence of Rab5 and Rab7 on phagosomes containing *T. whipplei* was further investigated by quantifying in immunofluorescence the colocalisation of *T. whipplei* with either Rab5 or Rab7. As shown in **Figure 3D**, first, *T. whipplei* phagosomes acquired Rab5 (approximately 65% of *T. whipplei* phagosomes colocalised with Rab5 within 15 minutes) and continued to be positive for Rab5 for prolonged periods (∼90% from 4 hours to 12 days after infection) (**Figure 3D**). Second, in parallel to the presence of Rab5, *T. whipplei* phagosomes acquired Rab7 in a progressive manner, and more than 80% of phagosomes expressed Rab7 by 4 hours (**Figure 3C**). Therefore, the *T. whipplei* fraction remaining at day 12 post-infection colocalised with both Rab5 and Rab7, suggesting that *T. whipplei* survives and replicates in chimeric phagosomes expressing early and late endosomal GTPases. Thus, *T. whipplei* might block the conversion of its phagosome into a phago-lysosome by interfering at the transition stage from Rab5-positive early phagosomes to Rab7-positive late phagosomes.

### *T. whipplei* does not alter PI(3)P recruitment to phagosome membranes

**Figure 4:**
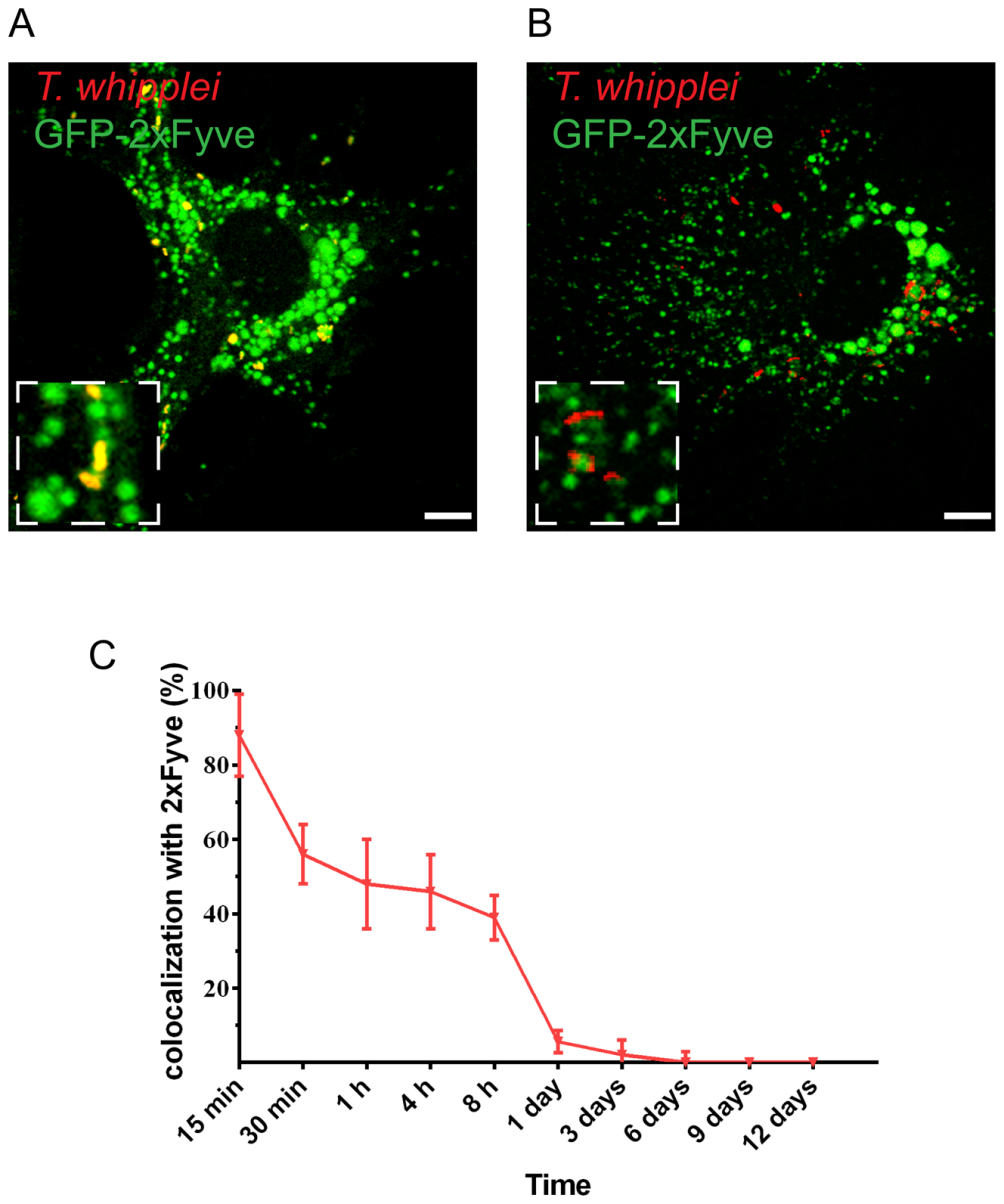
*T. whipplei* phagosomes transiently expressed PI(3)P. BMDMs were transfected with 2xFYVE-GFP using lentiviral vector and were infected with *T. whipplei*. The colocalisation of *T. whipplei* with 2xFYVE was analysed by immunofluorescence and confocal microscopy at 15 minutes (**A**) or 8 hours (**B**) after infection. (**C**) The percentage of *T whipplei* colocalising with 2xFYVE-GFP was quantified over time. The results are expressed as the mean ± SD from 3 experiments. The scale bars indicate 5 µm.

PI(3)P, the hallmark lipid on early endosomes, is involved in the coordination of the recruitment of Rab5 at the phagosome membrane [8], [23], and its production can be regulated by bacterial pathogens, such as *M. tuberculosis* [6]. We next asked whether a change in PI(3)P recruitment at the phagosome membrane could account for the persistence of Rab5 at the phagosome membrane. We investigated the presence of PI(3)P at the surface of *T. whipplei* phagosomes using BMDMs expressing a GFP-2xFYVE construct. FYVE domains are known to recognise PI(3)P with great selectivity and considerable affinity [24]; therefore, their presence on endosomes or phagosomes indicates PI(3)P production at these sites. We found that *T. whipplei* quickly colocalised with GFP-2xFYVE, and the timing of acquisition is similar to that observed for Rab5. At 15 min after infection with *T. whipplei*, more than 88% of *T. whipplei* phagosomes were surrounded by GFP-2xFYVE (**Figures 4A and 4C**). Nonetheless, this colocalisation is transient and undetectable thereafter (**Figures 4B and 4C**). The recruitment of PI(3)P to the *T. whipplei* phagosome is similar to that found for the LBC [22]; therefore, it may have a role in the initial recruitment of Rab5 on early phagosomes. However, it is unlikely that PI(3)P recruitment is responsible for Rab5 stabilisation and the defective Rab5 to Rab7 switch at the surface of the *T. whipplei* phagosome.

### *T. whipplei* blocks Rab5 function

**Figure 5:**
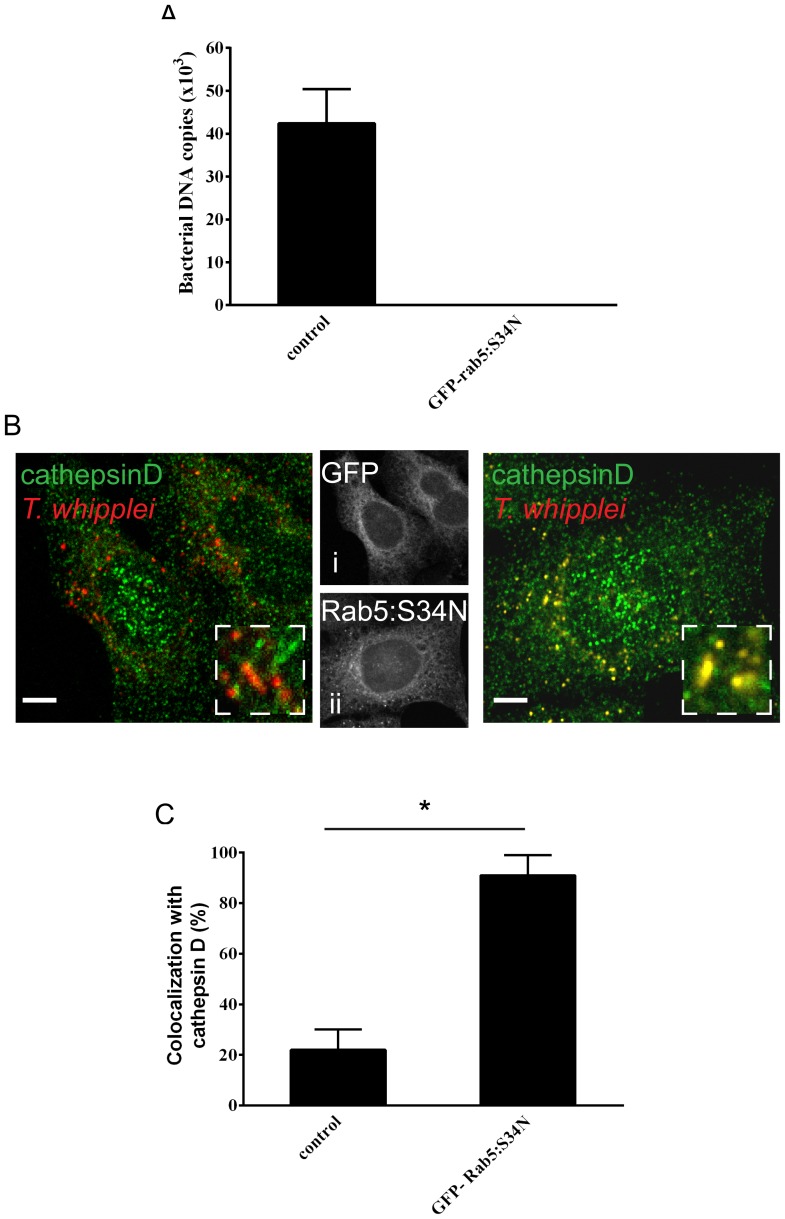
Overexpression of Rab5:S34N bypassed the pathogen-induced blockade of phago-lysosome biogenesis. BMDMs were incubated with *T. whipplei* for 4 hours and then washed to remove free bacteria. Infected BMDMs were transfected with GFP-Rab5:S34N or GFP (control) and incubated for additional periods. (**A**) At day 12 post-infection, the copy number of bacterial DNA was evaluated by qRT-PCR. The results are expressed as the mean ± SD from 3 experiments. (**B**) The colocalisation of *T whipplei* with cathepsin D was analysed in BMDMs expressing GFP (left panel) or GFP-Rab5:S34N (right panel) by immunofluorescence and confocal microscopy (at day 3 post-infection). (i) and (ii) show the expression patterns of GFP and GFP-Rab5:S34N. The scale bars indicate 5 µm. (**C**) The percentage of *T whipplei* colocalising with cathepsin D was quantified (at day 3 post infection). The results are expressed as the mean ± SD from 3 experiments (*p<0.05).

Rab5 and Rab7 continuously shift from active, GTP-bound forms to inactive, GDP-bound forms. The GTP forms are necessary for the recruitment of effectors, which promote and control fusion events; the conversion to the GDP forms allows the progression towards later stages of phagosome maturation [25]. Rab5 inactivation and depletion from endosomal membranes and Rab7 recruitment to early endosomes are concomitant and interlinked events [3], [4]. Because Rab5 and Rab7 coexist on *T. whipplei* phagosomes, we hypothesised that Rab5 is blocked in the active, GTP-bound form, leading to the blockade of the Rab cycle and the absence of progression towards a late phagosome. Therefore, in 4-hour-infected BMDMs, we overexpressed GFP-Rab5:S34N, a dominant-negative mutant of Rab5, which is locked in the inactive, GDP-bound form and therefore might complement the defect in the progression toward late compartments. Remarkably, we found that in this condition, the blockade of phagosome maturation was bypassed. *T. whipplei* was eliminated (**Figure 5A**): at 12 days post-infection, *T. whipplei* DNA was undetectable compared to control (∼40×10^3^ *T. whipplei* DNA copies), and more than 90% of the pathogen colocalised with cathepsin D at day 3, indicating transport to the phago-lysosomes (**Figures 5B and C**).

In sum, these results suggest that *T. whipplei* impairs the GTPase cycle of Rab5 and its depletion from the phagosomal membranes, blocking the transition from early to late phagosomes.

## Discussion

*T. whipplei* inhibits phago-lysosome biogenesis to create a suitable niche in its host cells for its replication. However, the mechanisms used by this pathogen to subvert phago-lysosome maturation have remained unknown. In this paper, we used biochemical and cell biological approaches to better characterise the intracellular compartment where *T. whipplei* survives and replicates, showing that it is a phagosome expressing the two critical GTPases Rab5 and Rab7 in addition to Lamp-1. Overexpression of the inactive, GDP-bound form of Rab5 bypassed the blockade of phago-lysosome biogenesis induced by the pathogen, suggesting that *T. whipplei* inhibits the switch in the transition from early to late phagosomes by interrupting the Rab5 GTPase cycle. Our results provide new molecular insights into the mechanisms evolved by *T. whipplei* to subvert phago-lysosome biogenesis in macrophages.

Several microorganisms are known to block phagosome conversion by acting on Rab proteins or Rab effectors [13]. *M. tuberculosis* blocks the conversion of the phagosome at the Rab5 stage; however, markers of the later stages of phagosome maturation, such as Rab7, were not found [26]. Similarly, the phagosome containing *H. pylori* is positive for Lamp-1 and Rab7 but not for either lysosomal enzymes or earlier markers [5]. To our knowledge, no microorganism has previously been described to induce a “chimeric” phagosome that stably expresses both Rab5 and Rab7, markers of early and late phagosomes. EEA1, one of the Rab5 downstream effectors, which is known to label a subpopulation of endosomes [27], [28], did not colocalize with *T. whipplei*, suggesting that *T. whipplei* accumulates in a subset of Rab5 positive phagosomes. Moreover, we excluded the inhibition of PI(3)P production as mechanism for Rab5 inactivity because cells expressing 2xFYVE-GFP did not show any stabilisation of this lipid in the *T. whipplei* compartment. Therefore, the mechanism used by *T. whipplei* to subvert phagosomes appears to be novel and specific for this pathogen, opening new perspectives towards the understanding of Whipple's disease and the establishment of novel diagnostic and therapeutic tools.

The transition from early to late phagosomes is highly correlated to the transition from Rab5 to Rab7 [29]. Rab5 and Rab7 co-exist only for a few minutes at the membrane of the compartments, and then, Rab5 inactivation and depletion from the membranes allows for conversion to active Rab7 and progression toward lysosomes [3], [4], [21], [22]. Overexpression of the inactive, GDP-bound form of Rab5 (Rab5:S34N) allowed the maturation of the phagosome containing *T. whipplei*, which fuses with a lysosome and become a phago-lysosome within which the pathogen is killed. Therefore, we hypothesised that *T. whipplei* affects Rab5 activity, leading to its accumulation in the active form (Rab5:GTP) at the surface of phagosome. The absence of the transition from Rab5:GTP to Rab5:GDP inhibits the switch from Rab5 to Rab7, despite the recruitment of Rab7 at the phagosome membrane because only the interruption of Rab5 activity allows the Rab7 function to take over and allows maturation to progress towards later compartments. The HOPS complex and SAND-1/Mon1 have been described as crucial regulators of the switch from Rab5 to Rab7 [4], [21], [22]. However, the complexity of the Rab machineries implies a complex regulatory mechanism for endosome and phagosome maturation. It would be interesting to use the *T. whipplei* chimeric phagosomes as tools to screen and identify factors implicated in the regulation of Rab5 and Rab7 activity.

**Figure 6:**
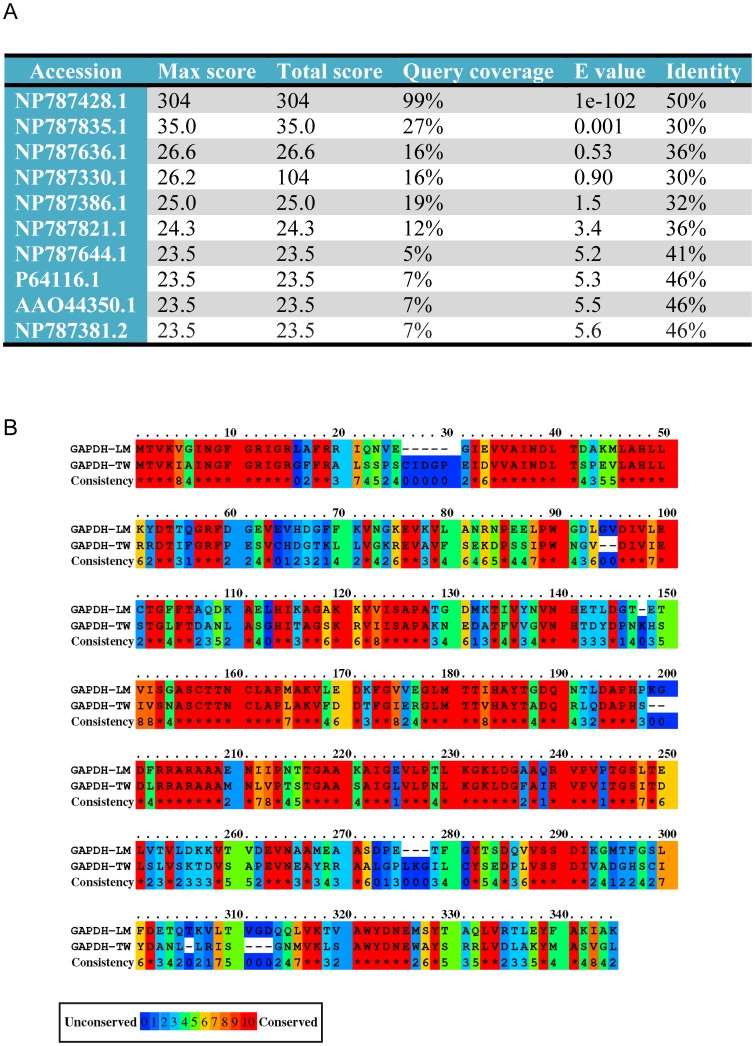
*T. whipplei* codes for an orthologue of GAPDH-LM. (**A**) Results of the Protein BLAST between glyceraldehyde-3-phosphate dehydrogenase from *L. monocytogenes* (GAPDH-LM, NP465982) and the genome of *T. whipplei strain Twist (taxid:203267)*. (**B**) Protein-protein alignment between glyceraldehyde-3-phosphate dehydrogenase (GAPDH-LM, NP465982) from *L. monocytogenes* and GAPDH from *T. whipplei* (GAPDH-TW). The results are colour-coded for amino acid conservation. The conservation scoring was performed by PRALINE (http://www.ibi.vu.nl/programs/pralinewww/). The scoring scheme works from 0, for the least conserved alignment position, to 10, for the most conserved alignment position.

Interestingly, *L. monocytogenes* also blocks the transition from Rab5-positive phagosomes before it escapes into the cytosol through glyceraldehyde-3-phosphate dehydrogenase (GAPDH-LM, NP465982), which induces Rab5a-specific ADP ribosylation and blocks the GDP/GTP exchange activity [11] [30]. A bio-informatic analysis of the *T. whipplei* genome revealed that *T. whipplei* encodes a protein with 50% identity and 99% coverage (E value 2e^−102^) at the protein level with the GAPDH-LM (**Figures 6A and 6B**). This protein is annotated as a *T. whipplei* GAPDH (GAPDH-TW, NP787428.1). We also found a GAPDH-LM homologue in *Tropheryma whipplei* (taxid:2039) and *Tropheryma whipplei* TW08/27 (taxid:218496) (not shown). Currently, there are no tools available to generate genetic modifications of *T. whipplei* DNA and to invalidate the GAPDH-TW function. Therefore, whether *T. whipplei* GAPDH (GAPDH-TW) interferes with Rab5 GTPase activity remains a very interesting and open question.

In conclusion, we reveal here that by interfering with Rab5 activity, *T. whipplei* creates a chimeric Rab5 and Rab7 compartment unable to fuse with lysosomes in which the pathogen can survive and replicate.

## Materials and Methods

### Ethics Statement

All of the animal experiments were conducted according to the Guiding Principles of Animal Care and Use defined by the Ethics Committee for animal experimentation (N°14 from the National Study Committee on Ethics of Animal Experimentation), according to the rules of Decret N°87–848 as of 10/19/1987. All of the animal experiments were also approved by the Ethics Committee for animal experimentation (N°14 from National Study Committee on Ethics of Animal Experimentation) at the institution where the experiments were performed (Faculty of Medicine, Marseille, experimentation permit number 10-300122013).

### Antibodies and reagents

Antibodies specific for Rab5 (Ab13253) as well as EEA1 (Sigma), active cathepsin D (CTD19), Rab7 (R4779) and Lamp-1 (1D4B) were purchased from Abcam, Sigma and DSHB. Secondary antibodies were purchased from Invitrogen.

### Bacteria

The *Twist*-Marseille strain of *T. whipplei* (CNCM I-2202) was cultured within HEL cells (CCL-37; American Type Culture Collection) and purified as described previously [31]. Bacteria were counted by immunofluorescence, and their viability was assessed using the Live and Dead BacLight bacterial viability kit (Invitrogen) [16].

### Cell culture

Mouse Bone Marrow-Derived Macrophages (BMDMs) were isolated and prepared as previously described [32].

### Quantitative real-time PCR (qRT-PCR)

BMDMs (10^5^ cells/assay) were infected with *T. whipplei* as previously described [15]. BMDMs were lysed, and DNA was extracted using a QIAamp DNA MiniKit (Qiagen). PCR was performed using a LightCycler-FastStart DNA Master SYBR Green system (Roche) and was conducted with primers specific for the *T. whipplei* 16S-23S ribosomal intergenic spacer region (tws3f and tws4r), as described previously [33]. In each PCR run, a standard curve was generated using serial dilutions ranging from 10 to 10^8^ copies of the intergenic spacer region and established by the LightCycler 5.32 software (LC-Run version 5.32; Roche) as previously described [15].

### Phagosome purification

BMDMs (10^8^ cells) were incubated for 1 hour with latex beads (1 µm, fluorescent red, Sigma-Aldrich) at a 1/5000 dilution or for 4 hours with *T. whipplei* (bacterium-to-cell ratio: 50∶1). Then, BMDMs were washed to remove free particles or bacteria, and they were incubated for 12 days in RPMI 1640 containing 10% FCS. The compartments containing latex beads (LBC) or *T. whipplei* (TwC) were purified according to the procedure described by Desjardins et al. [18]. Briefly, BMDMs were washed in cold PBS containing protease inhibitors (Complete, Roche) and scraped with a rubber scraper at 4°C. The cells were pelleted, homogenised, and washed in homogenisation buffer (250 mM sucrose, 3 mM imidazole, pH 7.4) containing protease inhibitor (Complete, Roche) at 4°C. Then, they were resuspended in 1 ml of homogenisation buffer containing protease inhibitor and homogenised on ice in a cell homogeniser. Unbroken cells were pelleted and centrifuged at 1200 rpm for 5 min, and the supernatant (PNS) was recovered and deposited on a sucrose gradient, as described in Figure 1. The LBC and TwC were then isolated on a sucrose step gradient (all sucrose solutions are wt/wt in 3 mM imidazole, pH 7.4) [18]. LBC and TwC were stored at −80°C after snap freezing in liquid nitrogen, in PBS with protease inhibitor.

### Western blotting

Phagosomes were analysed by immunoblotting (15% SDS polyacrylamide gels). The blots were then visualised using Immobilon Western Chemiluminescent HRP substrate (Millipore).

### Immunofluorescence and confocal microscopy

Infected cells were fixed with 3% paraformaldehyde in phosphate-buffered saline (PBS pH 7.4) and prepared for immunofluorescent labelling, as previously described [34]. Coverslips were mounted in Mowiol, and slides were viewed on an inverted Leica TCS SPE confocal laser-scanning microscope (Leica, Heidelberg, Germany). Image acquisition was performed using Leica Confocal Software. The collected images were processed using Adobe Photoshop CS5. The cells were evaluated as follows: 25 fields with at least three cells per field were examined for each experimental condition; in total, approximately 100 cells were examined per experimental condition. Each cell contained at least 3 to 5 phagosomes. In total, more than 400 phagosomes were examined per experimental condition. The colocalisation analysis was performed using ImageJ software (http://rsb.info.nih.gov/ij) and the JaCoP plugin (http://rsbweb.nih.gov/ij/plugins/track/jacop.html), as previously described [35], [36]. The calculation of overlap is based on the calculation of the Manders coefficients M1 and M2, reflecting channel1/channel2 overlap and channel2/channel1 overlap.

### Cell transfection

BMDMs were transiently transfected using lentiviral technology (Invitrogen) as previously described [34]. Briefly, GFP-Rab5:S34N and GFP-2xFYVE were amplified from pEGFP-Rab5:S34N and pEGFP-2xFYVE vectors, respectively. Then, GFP, GFP-Rab5:S34N and GFP-2xFYVE were subcloned into the entry clone pENTR TOPO vector using the TOPO cloning system, as recommended by the manufacturer (Invitrogen). The inserted DNA was transferred into the destination vector to create a lentivirus encoding GFP, GFP-Rab5:S34N, GFP-2FYVE using the ViraPower HiPerform Gateway Expression System (Invitrogen), as recommended by the manufacturer. Viruses were produced using 293T cells, and viral supernatants were harvested 48 and 72 hours after transfection. The supernatants were centrifuged at 1,600×*g* for 15 minutes at 4°C, filtered through 0.45-µm filters, collected and concentrated using PEG-*it* virus precipitation solution (System Biosciences), and then stored at −80°C.

### RT-PCR

RNA was extracted from infected BMDMs using the QIAamp RNA MiniKit (Qiagen). cDNA was synthesized from 1 µg of total RNA using SuperScript II RNase H reverse transcriptase (Invitrogen). Specific primers for each gene (actin 5′tggaatcctgtggcatccatgaaac, actin 3′taaaacgcagctcagtaacagtccg, Rab5 5′cgggccaaatactggaaata, Rab5 3′ aggacttgcttgcctttgaa, Rab7 5′gagcggactttctgaccaag, Rab7 3′ccggtcattcttgtccagtt) were designed using Primer3Plus. PCR was performed using HotStar *Taq* polymerase (Qiagen) following manufacturer's recommendations, and PCR products were electrophoresed on 1% agarose gel containing ethidium bromide. Data were acquired with Gel Doc 2000 (BioRad) and gene expression was normalized to the β-actin gene.

### Statistical analysis

The results are expressed as the mean ± SD and were analysed using the non-parametric Mann-Whitney *U* test. Differences were considered significant at *p*<0.05.

## Supporting Information

Figure S1 ***T. whipplei*** **did not modulate the transcription of Rab5 and Rab7 genes.** BMDMs were infected with *T. whipplei* (bacterium-to-cell ratio of 50∶1) for different periods. The amount of Rab5 and Rab7 mRNAs was analysed by RT-PCR and electrophoresis on agarose gel. The micrograph is representative of 3 experiments.
(TIF)
