# Supplementary figures and images for: Tropheryma whipplei, the Agent of Whipple's Disease, Affects the Early to Late Phagosome Transition and Survives in a Rab5- and Rab7-Positive Compartment
Source: PLoS One. 2014 Feb 24;9(2):e89367. doi: 10.1371/journal.pone.0089367 (PMC3933534; doi:10.1371/journal.pone.0089367)

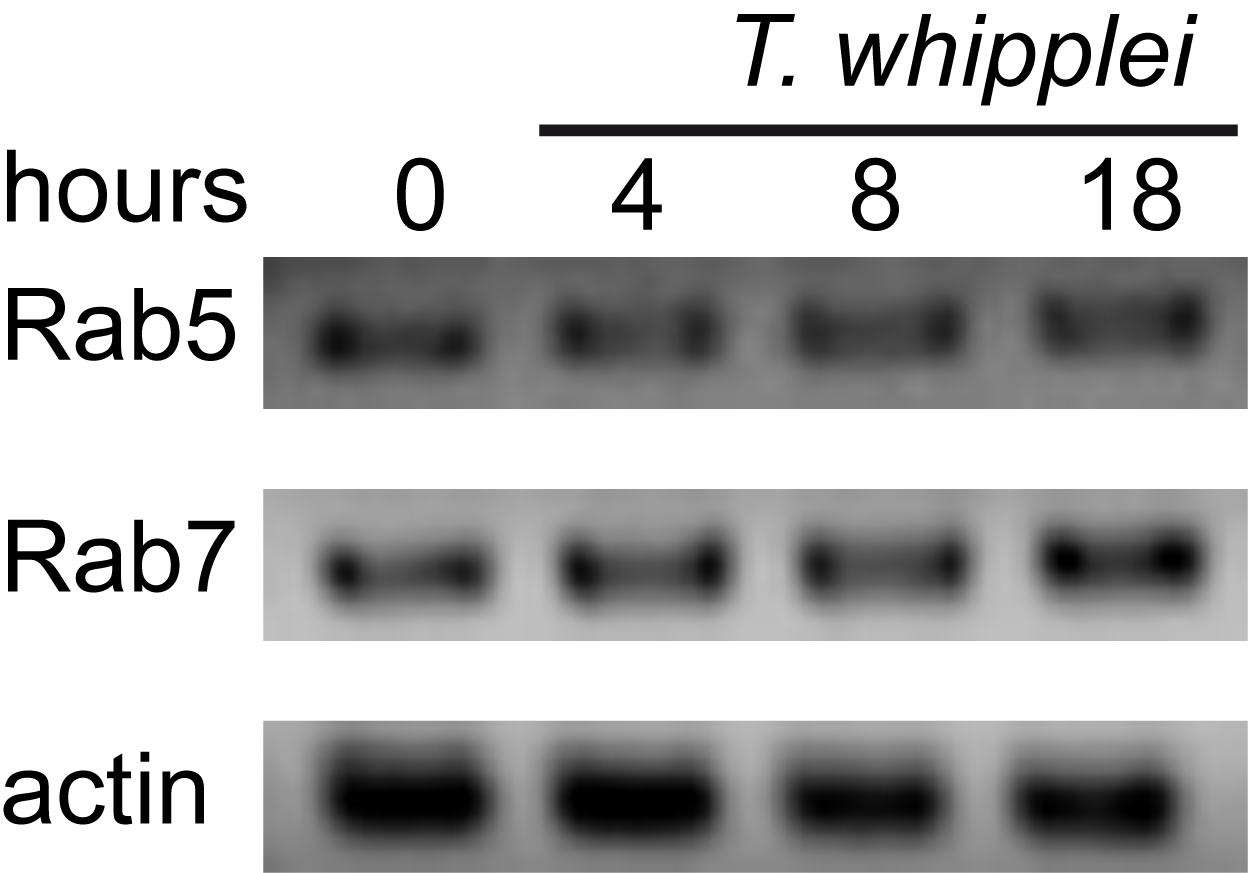

Supplement: Figure S1 — T. whipplei did not modulate the transcription of Rab5 and Rab7 genes. BMDMs were infected with T. whipplei (bacterium-to-cell ratio of 50∶1) for different periods. The amount of Rab5 and Rab7 mRNAs was analysed by RT-PCR and electrophoresis on agarose gel. The micrograph is representative of 3 experiments. (TIF) [file pone.0089367.s001.tif]
